# Cisplatin Induces Differentiation of Breast Cancer Cells

**DOI:** 10.3389/fonc.2013.00134

**Published:** 2013-06-03

**Authors:** Praseetha Prabhakaran, Foteini Hassiotou, Pilar Blancafort, Luis Filgueira

**Affiliations:** ^1^School of Anatomy, Physiology and Human Biology, The University of Western Australia, Crawley, Perth, WA, Australia; ^2^Faculty of Biosciences and Bioengineering, Universiti Teknologi Malaysia, Skudai, Johor, Malaysia; ^3^School of Chemistry and Biochemistry, The University of Western Australia, Crawley, Perth, WA, Australia; ^4^Department of Medicine, University of Fribourg, Fribourg, Switzerland

**Keywords:** breast cancer cells, cancer stem cells, cisplatin, proliferation, differentiation

## Abstract

Breast tumors are heterogeneous including cells with stem cell properties and more differentiated cells. This heterogeneity is reflected into the molecular breast cancer subtypes. Breast cancer stem cells are resistant to chemotherapy, thus recent efforts are focusing on identifying treatments that shift them toward a more differentiated phenotype, making them more susceptible to chemotherapy. We examined whether the drug cisplatin induces differentiation in breast cancer cell lines that represent different breast cancer subtypes. We used three cell lines representing triple-negative breast cancers, BT-549 and MDA-MB-231 (claudin-low), and MDA-MB-468 (basal-like), along with estrogen and progesterone receptor positive MCF-7 cells (luminal). Cisplatin was applied at 2.5, 5, 10, and 20 μM, and cell viability and proliferation were measured using MTS and BrdU assays, respectively. The effect of cisplatin on the cellular hierarchy was examined by flow cytometry, immunofluorescence and qRT-PCR. Cisplatin treatment of 10 and 20 μM reduced cell viability by 36–51% and proliferation capacity by 36–67%. Treatment with cisplatin resulted in 12–67% down-regulation of stem cell markers (CD49f, SSEA4) and 10–130% up-regulation of differentiation markers (CK18, SMA, β-tubulin). At the mRNA level, CD49f was down-regulated whilst β-tubulin was up-regulated in the claudin-low cell lines. SSEA4 protein expression decreased upon cisplatin treatment, but SSEA4 mRNA expression increased indicating a differential regulation of cisplatin at the post-transcriptional level. It is concluded that cisplatin reduces breast cancer cell survival and induces differentiation of stem/progenitor cell subpopulations within breast cancer cell lines. These effects indicate the potential of this drug to target specific chemotherapy-resistant cells within a tumor.

## Introduction

Breast cancer is one of the most frequent cancers among women worldwide (Jemal et al., 2010). Much of the difficulty in treating this disease is due to the heterogeneity of breast tumors, which consist of a cellular hierarchy similar to the normal breast (Villadsen et al., 2007; Visvader, 2009; Hassiotou and Geddes, 2012; Hassiotou et al., 2012; Hassiotou et al., 2013a,b), from cancer cells with stem cell properties to more differentiated tumor cells (Prat et al., 2010). Breast cancer stem-like cells (BCSCs) comprise a cell subpopulation within a tumor that is responsible for the initiation, progression, chemotherapy resistance, and metastasis of the tumor (Clarke et al., 2006; Croker and Allan, 2008; Short and Curiel, 2009; Monteiro and Fodde, 2010; Perou, 2011; Zhao et al., 2011; Dave et al., 2012; Sampieri and Fodde, 2012). BCSCs originate from normal mammary stem cells (MaSCs) that have become tumorigenic due to multiple genetic and epigenetic changes (Wicha et al., 2006; Shafee et al., 2008). Possessing similar properties to normal MaSCs, BCSCs proliferate and undergo multi-lineage differentiation, resulting in the growth and heterogeneous histological appearance of breast tumors (Turashvili et al., 2007; Levina et al., 2008).

Breast cancer heterogeneity and cellular hierarchy has led to the identification of five molecular subtypes, which are distinguished based on their molecular and clinical characteristics, and pathogenesis (Sotiriou and Pusztai, 2009; Bosch et al., 2010; Hastak et al., 2010; Al-Ejeh et al., 2011). These include the poorly characterized claudin-low tumors, the basal-like, human epidermal growth factor receptor 2 (HER2) positive, luminal A, and luminal B tumors.

Luminal breast tumors, represented *in vitro* by MCF-7 cells, are more differentiated and are often successfully treated with chemotherapy, indicating that more differentiated tumors are more susceptible to treatments. In contrast, the basal-like (e.g., MDA-MB-468 cells) and claudin-low subtypes (e.g., MDA-MB-231 cells) are less differentiated, difficult to treat with poor prognosis (Hastak et al., 2010; Holliday and Speirs, 2011). Often, basal-like and claudin-low tumors lack the estrogen (ER), progesterone (PR), and HER2 receptors, and are thus triple-negative (Hastak et al., 2010; Holliday and Speirs, 2011; Tiwary et al., 2011; Byrski et al., 2012). These tumors are fueled by BCSCs, are highly resistant to chemotherapy (Hastak et al., 2010; Tiwary et al., 2011), and are very proliferative with worst survival rates (Bosch et al., 2010; Hastak et al., 2010; Tiwary et al., 2011). Thus, recent efforts have been focusing on treatments that may shift the less differentiated BCSCs toward a more differentiated phenotype, making them more susceptible to treatment options, and eliminating the chance for recurrence and/or metastasis.

Cisplatin (cis-diamminedichloroplatinum II) is a metal-based anti-cancer drug (Rosenberg et al., 1965, 1969) that has been used extensively in the past four decades for the treatment of many cancers (Nishiyama et al., 2003; Dickson et al., 2011), including breast, testicular, ovarian, cervical, head and neck, and small cell lung cancers (Basu and Krishnamurthy, 2010; Florea and Busselberg, 2011; Pines et al., 2011). Particularly in breast cancer, cisplatin has been used in combination with other drugs, such as taxanes, vinca alkaloids, and 5-fluorouracil (Florea and Busselberg, 2011; Holliday and Speirs, 2011), resulting in synergistic or additive effects. Cisplatin is known to cause DNA damage by forming Pt-DNA adducts at the 1,2-intrastrand crosslink, leading to the activation of various signal transduction pathways (Zeidan et al., 2008; Basu and Krishnamurthy, 2010; Florea and Busselberg, 2011; Wang et al., 2011). However, its exact mechanism of action and specificity are still not well established. To give insight into the mechanisms through which cisplatin sensitizes breast cancer cells to chemotherapy, we examined the effects of cisplatin on cell phenotype and survival using four human cancer cell lines representing different molecular and differentiation subtypes of breast cancer.

## Materials and Methods

### Cell culture

BT-549, MDA-MB-231, MDA-MB-468, and MCF-7 cells (American Type Culture Collection, ATCC) (Table 1) were cultured in T25 flasks (Corning, Tewksbury, MA, USA) at 37 °C and 5% CO_2_ in DMEM/F12 + glutamax ™1 (Invitrogen, Carlsbad, CA, USA), supplemented with 20% fetal bovine serum (FBS) (Serana, WA Pty Ltd., Bunbury, WA, Australia), and 1% antibiotic-antimycotic (Invitrogen). BT-549 and MDA-MB-231 cells were passaged twice a week, whilst MDA-MB-468 and MCF-7 once a week at 60–70% cell confluency.

**Table 1:** **Breast cancer cell lines used**.

Cell line	Tumor type	Tumor classification	State of differentiation
BT-549	Papillary invasive ductal carcinoma	Claudin-low	Less differentiated
MDA-MB-231	Adenocarcinoma	Claudin-low	Least differentiated
MDA-MB-468	Adenocarcinoma	Basal-like	Differentiated
MCF-7	Adenocarcinoma	Luminal A	Most differentiated

### Cytotoxicity and proliferation assays

Confluent cell cultures were used for experiments. About 3 × 10^3^ cells per 100-μL and per well were seeded in flat bottom 96-well plates (Sarstedt, Newton, USA). After 24 h, cisplatin was added at different concentrations (2.5, 5, 10, and 20 μM) (Sigma-Aldrich, St Louis, MO, USA) chosen according to the range used in treatments. After incubation for 24 h, cell viability was assessed by MTS colorimetric assay, using. Cell Titer 96 ^®^Aqueous (Promega, Madison, USA), according to the manufacturer’s instructions. Cell proliferation was measured using a BrdU colorimetric assay (Roche Diagnostics, Mannheim, Germany), according to the manufacturer’s instructions. Experiments were done in quadruplicate in three independent experiments.

### Immunofluorescence microscopy

Cells were grown on coverslips in 24-well plates (Sarstedt) for 24 h. Cisplatin at 20 μM was added and cells were incubated for 24 h. Cells were then fixed with 1% paraformaldehyde (PFA) in PBS/2% sucrose, permeabilized with 0.1% Triton in PBS for 30 min, incubated overnight with primary antibodies (Table 2), and then incubated for 4 h with secondary antibodies (Table 2) and DAPI (Roche, 1:100) for nuclear staining. Appropriate negative controls (secondary antibody only) were used. Cells were imaged using an Olympus 1X71 inverted optical microscope and an upright Nikon Eclipse 90i microscope.

**Table 2:** **Antibodies used**.

Antibody	Clone	Cat. number	Company	Application
Nestin	3k1	09-0045	Stemgent	FC: 1:50
CK18	CY90	MCA1864H	AbD Serotec	FC: 100 μL, IF: 100 μL
Smooth muscle actin (SMA)	CGA7	Sc-53015	Santa Cruz Biotechnology, Inc.	FC: 1:50, IF: 1:10
β-Tubulin	–	Hybridoma		FC: 100 μL, IF: 100 μL
Stage specific embryonic antigen-4 (SSEA4)	MC-813-70	09-0006	Stemgent	FC: 1:50
Integrin-α6 (CD49f)	GoH3	555735	BD Pharmingen™	FC: 1:4
Alexa Fluor 488 donkey anti-mouse IgG (H + L)	–	A-21202	Invitrogen, USA	FC: 1:50, IF: 1:100
Alexa Fluor 488 donkey anti-rabbit IgG (H + L)	–	A-21206	Invitrogen, USA	FC:1:50
FITC rat IgG_2_a.k	R35-95	555843	BD Pharmingen™	FC:1:4

### Flow cytometry

Confluent cultures of untreated and 20-μM cisplatin-treated breast cancer cells were passaged by trypsinization. A day later, adherent cells were gently scraped and centrifuged at 1200 rpm for 5 min. Cells were fixed in 1% PFA in PBS/2% sucrose for 20 min at room temperature, and incubated with primary antibodies (Table 2) for 1 h at 4 °C, followed by incubation with secondary antibodies (Table 2) for 30 min at 4 °C. All intracellular marker antibodies were prepared in permeabilization solution (0.05% Tween-20 in PBS), whilst surface marker antibodies were prepared in 7% FBS in PBS. Appropriate negative controls (secondary antibody only) were also used. Data acquisition was done with a FACS Calibur Flow Cytometer (Becton Dickinson, NJ, USA), and 10,000 events were collected and analyzed per sample. FlowJo was used for data analysis. Expression levels were analyzed as the standardized difference in the Mean Fluorescence Intensity (MFI) between the control and the test. A MFI threshold was set to distinguish the levels of protein expression: MFI ≤ 20 = very low; 21 ≤ MFI ≤ 40 = low; 41 ≤ MFI ≤ 60 = medium; 61 ≤ MFI ≤ 80 = high; 81 ≤ MFI ≤ 100 = very high; and MFI ≥ 101 = extremely high.

### Quantitative real-time polymerase chain reaction

Total cellular RNA was extracted using RNAzol^®^ RT (Molecular Research Center, Inc.). RNA quantity and quality were assessed with NanoDrop 1000 (NanoDrop, Wilmington, DE, USA). For each sample, 1 μg RNA was treated with RQ1 RNase-Free DNase (Promega, Madison, USA) and was reverse transcribed using MMLV (Promega) by incubating at 25 °C (10 min), 55 °C (50 min), and 70 °C (15 min) using PTC-100 ™Programmable Thermal Controller (MJ Research Inc.). The RT Reaction Clean-Up MoBio kit (MoBio Lab Inc., CA, USA) was used for cDNA clean up. The Brilliant SYBR green quantitative real-time polymerase chain reaction (qRT-PCR) Master Mix consisting of 5 μL of IQ ™SYBR ^®^Green Supermix (Bio-Rad, CA, USA), 1 μL of each forward and reverse primers for each gene (Table 3), 1 μL of H_2_O and 2 μL of cDNA were used to detect the relative abundance of transcripts. The conditions for all qRT-PCR reactions were as follows: 10 s at 95 °C followed by 30 s at 54, 55, and 60 °C (Table 3), and 15 s at 72 °C for 40 cycles. Validation was done by sequencing of the PCR products, analysis of the melting curves and use of β-actin as the positive control and non-template sample as the negative control.

**Table 3:** **Primers used for RT-PCR**.

Gene	Forward primer	Reverse primer	Annealing temperature (°C)
SSEA4	5′TGG ACG GGC ACA ACT TCA TC 3′	5′GGG CAG GTT CTT GGC ACT CT 3′	54
CD49f	5′ATG GAG GAA ACC CTG TGG CT 3′	5′ACG AGA GCT TGG CTC TTG GA 3′	60
β-Tubulin 6	5′AGG CTA CGT GGG AGA CTC G 3′	5′GCC CTG GGC ACA TAT TTC T 3′	60
β-Actin	5′CGG CAT TCA CGA AAC 3′	5′GGG CAG TGA TCT CTT 3′	55

### Statistical analysis

Statistical analysis and graphical exploration of the data were done in Microsoft Excel. The Student’s paired *t*-test with a two-tailed distribution was used to compare cisplatin-treated and untreated breast cancer cells. The results are presented as mean ± SD (MTS and BrdU) and mean ± SEM (flow cytometry and qRT-PCR), as indicated in the corresponding figure legends. The significance is shown as follows: *p ≤ 0.05; **p ≤ 0.0005; ***p < 0.0001.

## Results

### Cisplatin reduces viability and proliferation of breast cancer cells

**Figure 1:**
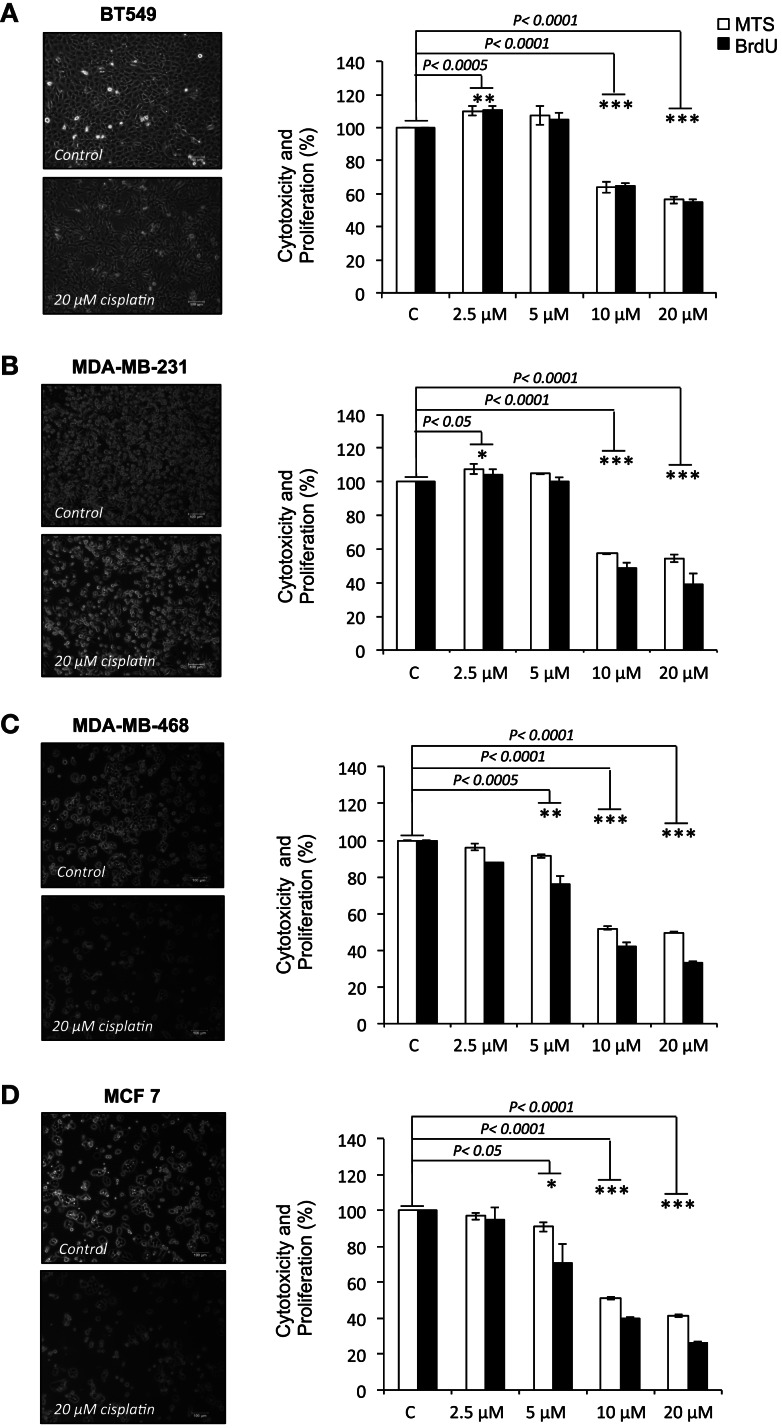
**Cisplatin reduces cell viability and proliferation in breast cancer cells**. **(A)** BT-549, **(B)** MDA-MB-231, **(C)** MDA-MD-468, and **(D)** MCF-7 breast cancer cells cultured without or with 20 μM cisplatin for 24 h. The bar charts show the effect of cisplatin at increasing concentrations (2.5, 5, 10, and 20 μM) on cell viability and proliferation in the BT-549, MDA-MB-231, MDA-MD-468, and MCF-7 respectively, which were determined by MTS (white bars) and BrdU (black bars) assays. Experiments were done in quadruplicate in three independent experiments. Bars are presented as mean ± SD (*n* = 3). *p ≤ 0.05; **p ≤ 0.0005; ***p ≤ 0.0001.

Cisplatin showed dose-dependent effects in all tested cell lines. At lower doses (2.5–5 μM), cisplatin enhanced cell viability and proliferation in BT-549 and MDA-MB-231 cells, whilst a small decline in viability and proliferation was observed in MDA-MB-468 and MCF-7 cells. At the doses most commonly reached in tissues in clinical treatments (10–20 μM), significant reductions in cell viability and proliferation were observed in all cell lines, but at different rates, ranging 36–51% for cell viability and 36–67% for proliferation (Figures 1A–D). MCF-7 cells, which are the most differentiated cells used, showed most dramatic reductions in cell viability at 10 and 20 μM cisplatin (49 and 58%, respectively; *p* < 0.0001), followed by MDA-MB-468 (48 and 51%, respectively; *p* < 0.0001), MDA-MB-231 (43 and 45%, respectively; *p* < 0.0001), and BT-549 (36 and 44%, respectively; *p* < 0.0001). Similarly, 5 μM caused significant reductions in cell viability and proliferation, but only in the more differentiated MDA-MB-468 (9 and 24%, respectively; *p* < 0.001) and MCF-7 (9 and 29%, respectively; *p* < 0.01). Cell proliferative capacity decreased more than cell viability in the cell lines at 10 and 20 μM: in MCF-7 60 and 74%, respectively (*p* < 0.0001), in MDA-MB-468 58 and 66%, respectively (*p* < 0.0001) and in MDA-MB-231 51 and 61%, respectively (*p* < 0.0001). Exception to this was BT-549 cells, which showed reduction in proliferation that was similar to the reduction of viability (36 and 45%, respectively; *p* < 0.0001), suggesting that the surviving cells may undergo differentiation. Upon cisplatin treatment, the less differentiated cells displayed a more differentiated phenotype (Figure 1B); BT-549 cells appeared more contracted and elongated (Figure 1A), and MDA-MB-231 cells appeared slightly enlarged and elongated (Figure 1B). Morphological changes were not as prominent in the more differentiated MDA-MB-468 and MCF-7 cells. These results demonstrate that cisplatin treatment caused a reduction in both cell viability and proliferation by interfering with cellular functions through unknown mechanisms, with the most prominent effects at the cisplatin concentrations that are expected in the clinical setting.

### Cisplatin induces breast cancer cell differentiation

**Figure 2:**
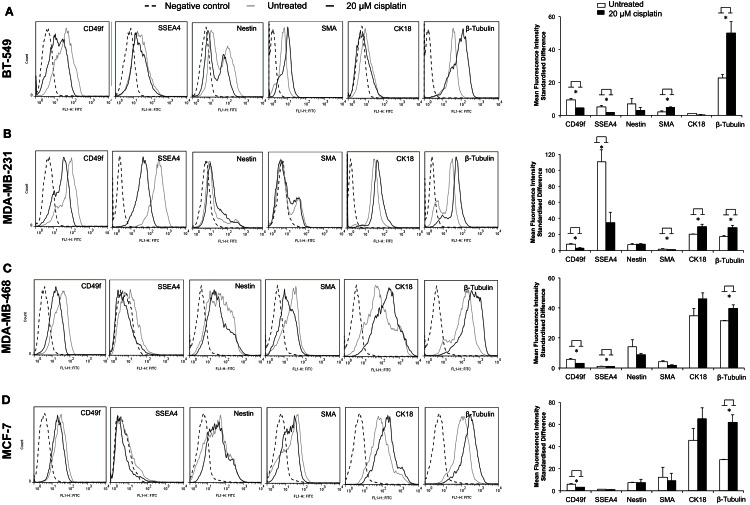
**Cisplatin shifts breast cancer cells toward a more differentiated phenotype**. Flow cytometric quantification of CD49f, SSEA4, nestin, SMA, CK18, and β-tubulin protein expression showed various levels in four breast cancer cell lines. **(A–D)** show flow cytometry histograms of expression of the above markers in BT-549, MDA-MB-231, MDA-MB-468, and MCF-7 (black dashed line: unstained control; gray line: FITC-stained untreated breast cancer cells; black line: FITC-stained 20 μM cisplatin-treated breast cancer cells). The bar charts show quantification of the level of expression based on the mean fluorescence intensity (MFI) standardized difference of cisplatin-treated (20 μM cisplatin for 24 h) and untreated BT-549, MDA-MB-231, MDA-MD-468, and MCF-7 breast cancer cells. Bars are presented as mean fluorescence intensity (MFI) standardized difference ± SEM (*n* = 3). *p ≤ 0.05.

The effect of cisplatin on expression of a variety of key markers, such as SSEA4, CD49f, nestin, SMA, CK18, and β-tubulin, was examined using flow cytometry. A cellular hierarchy was observed in the untreated cell lines (Figures 2A–D). Untreated BT-549, MDA-MB-231, MDA-MB-468, and MCF-7 cells expressed stem cell markers (SSEA4, CD49f, nestin), a myoepithelial marker (SMA), an epithelial marker (CK18) and a microtubule marker (β-tubulin). These markers were expressed at various levels depending on the differentiation status of each cell line (Figure 2; Table 4). BT-549 expressed medium to high levels of β-tubulin, CD49f, and nestin, but very low levels of SMA and CK18 (Figure 2; Table 4). MDA-MB-231 cells expressed medium to high levels of CK18, β-tubulin, CD49f, and SSEA4, whilst SMA and nestin were expressed at very low levels. In contrast, MDA-MB-468 cells and MCF-7 expressed CK18 at very high levels, whilst SSEA4, CD49f, and SMA were expressed at low levels (Table 4). Nestin was highly expressed in MDA-MB-468, but at low levels in MCF-7. β-Tubulin was expressed at medium and high levels, respectively, in these cell lines (Table 4).

**Table 4:** **Breast cancer cell characterization and effects of cisplatin on protein expression**.

Cell line	Cell type	Markers	No drug (MFI)	Cisplatin (MFI)	Up/down-regulation (%)
BT-549	Stem cells	SSEA4	22.2	7.2	↓(67.6)
		CD49f	60	27	↓(54.8)
	Progenitor cells	Nestin	45	21	↓(52.3)
	Differentiated cells	SMA	7.8	17.9	↑(129.5)
		CK18	7	2	↓(71.2)
		β-Tubulin	66	148.6	↑(125.2)
MDA-MB-231	Stem cells	SSEA4	518.2	205.3	↓(60.4)
		CD49f	53	24	↓(55)
	Progenitor cells	Nestin	35.8	45.5	↑(27)
	Differentiated cells	SMA	9.7	6.2	↓(36)
		CK18	41.2	45.4	↑(10.2)
		β-Tubulin	49.6	83.6	↑(68.5)
MDA-MB-468	Stem cells	SSEA4	6.5	4	↓(38.5)
		CD49f	27	15	↓(44.4)
	Progenitor cells	Nestin	81	65	↓(19.6)
	Differentiated cells	SMA	25	13	↓(48)
		CK18	161.8	262.4	↑(62.2)
		β-Tubulin	52.9	82	↑(55)
MCF-7	Stem cells	SSEA4	8.02	7.02	↓(12.5)
		CD49F	27.2	15.8	↓(41.9)
	Progenitor cells	Nestin	30.3	29.4	↓(3)
	Differentiated cells	SMA	14.2	10.2	↓(28.2)
		CK18	173.1	224	↑(29.4)
		β-Tubulin	74.9	141	↑(88.3)

MFI, mean fluorescence intensity; ESC, embryonic stem cell; SSEA4, stage specific embryonic antigen-4; CD49f, integrin subunit-α6; SMA, smooth muscle actin; CK18, cytokeratin 18.

**Figure 3:**
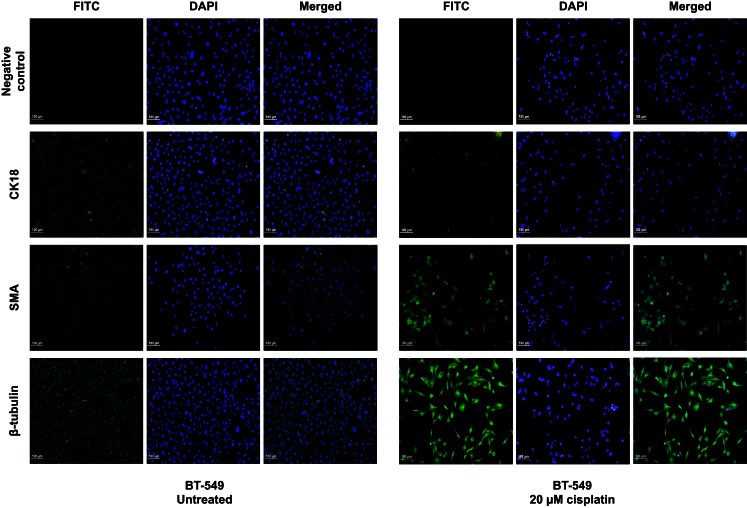
**Cisplatin influences expression of differentiation markers in BT-549 cells**. Differentiation markers SMA and β-tubulin were up-regulated upon treatment with 20 μM cisplatin in BT-549 cells. DAPI nuclear stain was used to stain the nucleus (blue), whilst all other markers are shown in green. Scale bars: 100 μm.

**Figure 4:**
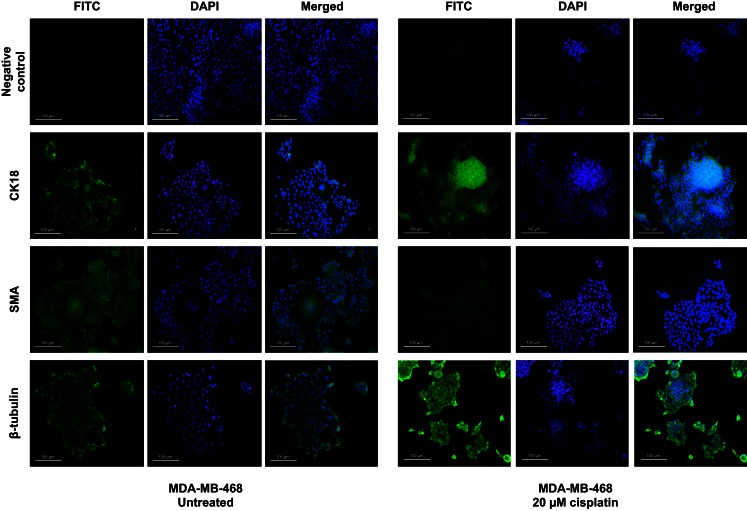
**Cisplatin influences expression of differentiation markers in MDA-MB-468**. Differentiation markers CK18 and β-tubulin were up-regulated upon treatment with 20 μM cisplatin in MDA-MB-468 cells. DAPI nuclear stain was used to stain the nucleus (blue), whilst all other markers are shown in green. Scale bars: 100 μm.

Treatment with cisplatin shifted the cellular hierarchy of these cell lines, causing distinct changes in cell phenotype and protein expression (Figure 2; Table 4). The stem cell markers SSEA4 and CD49f were significantly down-regulated in all the TNBC cell lines, BT-549 (*p* = 0.023 and *p* = 0.034, respectively), MDA-MB-231 (*p* = 0.018 and *p* = 0.036, respectively), and MDA-MB-468 (*p* = 0.03 and *p* = 0.018, respectively) (Figures 2A–C; Table 4). A significant reduction in CD49f expression was also observed in MCF-7 (*p* = 0.025), but a very small or negligible change in expression of SSEA4 (Figure 2D; Table 4). In addition to down-regulation of stem cell markers, cisplatin induced significant up-regulation of differentiation markers. β-Tubulin was up-regulated in MDA-MB-231 (*p* = 0.044), MDA-MB-468 (β-tubulin: *p* = 0.020), and MCF-7 (β-tubulin: *p* = 0.034), whilst CK18 expression increased significantly in MDA-MB-231 (*p* = 0.045), and only marginally in the latter two cell lines (Figures 2B–D; Table 4). In BT-549 cells, SMA (*p* = 0.014) and β-tubulin (*p* = 0.020) were up-regulated (Figure 2A; Table 4). Immunofluorescence imaging confirmed the increased expression of SMA, CK18, and β-tubulin upon cisplatin treatment (Figures 3 and 4). These results provided evidence of differentiation induction in the examined breast cancer cells upon cisplatin treatment.

### Differential gene regulation by cisplatin at the transcriptional and post-transcriptional levels

**Figure 5:**
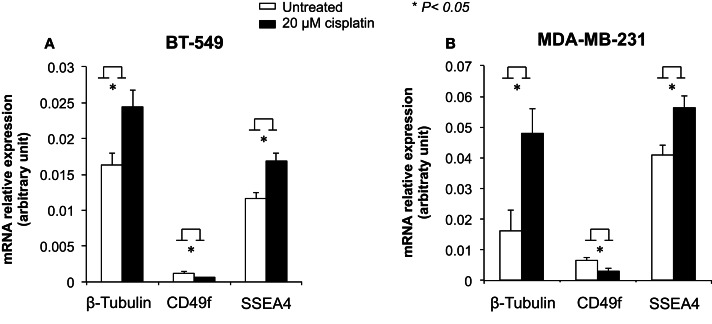
**Effects of cisplatin on mRNA expression of β-tubulin, CD49f, and SSEA4 in breast cancer cells**. Individual PCR reactions were normalized against internal positive control (β-actin) and plotted as the mRNA relative expression for both untreated and cisplatin-treated BT-549 **(A)** and MDA-MB-231 **(B)** breast cancer cells. Cells were incubated with 20 μM cisplatin for 24 h. Experiments were done in duplicates in three independent experiments. Bars are presented as mean ± SEM. *p ≤ 0.05.

**Figure 6:**
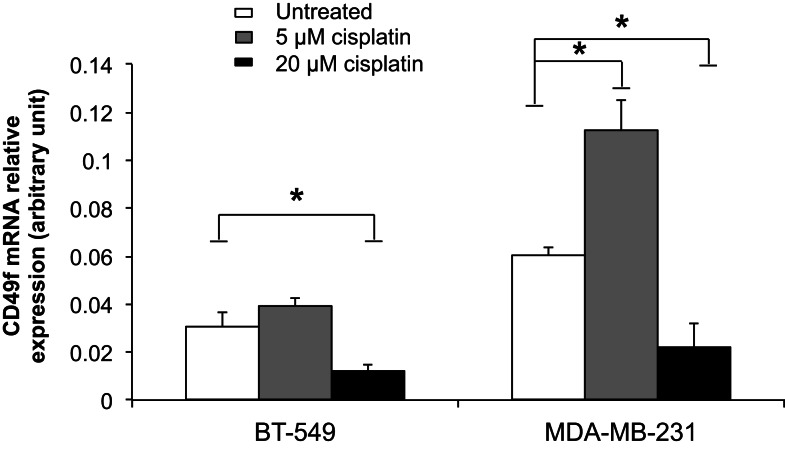
**Comparison of cisplatin effect on mRNA expression of CD49f, a stem cell marker, at low and high cisplatin doses in breast cancer cells**. Individual PCR reactions were normalized against internal positive control (β-actin) and plotted as the mRNA relative expression for both untreated and cisplatin-treated BT-549 and MDA-MB-231 breast cancer cells. Cells were incubated with 5 and 20 μM cisplatin for 24 h. Experiments were done in duplicates in three independent experiments. Bars are presented as mean ± SEM. *p ≤ 0.05.

To examine the role of cisplatin in gene regulation, CD49f, SSEA4, and β-tubulin mRNA expression levels were measured in 20 μM cisplatin-treated and untreated cells in two of the most invasive breast cancer cell lines, BT-549 and MDA-MB-231, which showed either low or very high protein expression levels prior to treatment, and were clearly differentiated by cisplatin. The mRNA expression of β-tubulin was significantly increased upon cisplatin treatment in both BT-549 (*p* = 0.043) and MDA-MB-231 (*p* = 0.021) (Figure 5). mRNA expression of CD49f significantly decreased with cisplatin treatment (*p* = 0.036 and *p* = 0.034, respectively) (Figure 5), whilst at lower cisplatin dose (5 μM) an increase in the mRNA expression of CD49f was observed (MDA-MB-231: *p* = 0.014) (Figure 6). The mRNA expression pattern for both β-tubulin and CD49f was consistent with the protein expression pattern. However, SSEA4 mRNA expression was significantly up-regulated in cisplatin-treated BT-549 (*p* = 0.019) and MDA-MB-231 cells (*p* = 0.022) (Figure 5), which was contrary to the protein expression pattern. This suggests that cisplatin may differentially regulate gene expression, with potential post-transcriptional effects for certain genes.

## Discussion

Recent efforts are focusing on the development of breast cancer treatments that specifically target the cancer stem cells (CSCs), which are responsible for tumor progression, metastasis, and recurrence. Given the resistance of CSCs to chemotherapy, successful treatments must first induce CSC differentiation to make them more susceptible to the killing effects of anti-cancer drugs. Here, we demonstrate cisplatin-inducted differentiation of common cell lines representing different subtypes of breast cancer, including TNBCs, which are the most aggressive and highly populated by CSCs. This effect of cisplatin was delivered via down-regulation of the stem cell markers CD49f and SSEA4, and subsequent cytotoxicity resulting in marked reduction in cell viability and proliferation. These findings give insight into the cellular hierarchy of breast tumors and suggest a novel mechanism of action for cisplatin that first differentiates and then kills breast cancer cells.

Cytotoxicity assays of cisplatin treatment revealed a cytotoxic effect a high doses (10 and 20 μM). The observed increase in cell proliferation at low cisplatin doses suggests that the CSCs, which are the most proliferative within a tumor, are not killed, and this needs to be taken into consideration in future cisplatin treatments. At high doses, cisplatin probably kills the tumor cells by interfering with cellular structure and function at the DNA level (Basu and Krishnamurthy, 2010), as has been shown previously. At the same time, cisplatin appeared to regulate gene expression, and therefore interfere with the stage of cellular differentiation, both at the mRNA and protein levels. Interestingly, the response to cisplatin treatment varied between the different cell lines tested, and for the different cisplatin doses examined. This is in agreement with previous studies showing that some tumor cells require high cisplatin doses, whilst other tumor cells require small doses to be killed (Foroodi et al., 2009; Wang et al., 2011; Milrot et al., 2012). Low, non-toxic dosage may have a different effect without causing cell death. This differential response suggests that cisplatin may act in a tumor-specific manner, depending on the properties and cellular hierarchy manifested by each tumor. This led us to investigate how cisplatin affects the cellular hierarchy and phenotypes of breast cancer cell lines.

Flow cytometric analysis of protein expression revealed a novel cellular hierarchy in the breast cancer cell lines examined which differed depending on the cell line. This was consistent with the previously suggested breast cancer heterogeneity (Clarke et al., 2006). The stem cell markers SSEA4 and CD49f were highly expressed in the more aggressive CSC-enriched and triple-negative cell lines, whereas the differentiation markers CK18 and β-tubulin were highly expressed in the more differentiated cell lines. It is of interest that the MDA-MB-468, a basal-like triple-negative line, is more differentiated than other triple-negative lines, such as BT-549 and MDA-MB-231.

Cisplatin treatment shifted this cellular hierarchy toward more differentiated cells by selectively targeting and down-regulating the stem cell markers CD49f and SSEA4 by 50–70% in the more invasive breast cancer cell lines (BT-549 and MDA-MB-231), whilst up-regulating the differentiation markers CK18, SMA, and β-tubulin by 10–130% (Figure 2; Table 4). High CD49f and SSEA4 expression has been associated with low levels of tumor differentiation and reduced survival in breast cancer patients, and is often more prevalent in TNBCs (Zeidan et al., 2008; Stagg and Pommey, 2009; Meyer et al., 2010; Sanges and Cosma, 2010). CD49f and SSEA4 along with other surface markers, such as CD24, CD44, CD133, have been commonly used for the detection of CSCs in solid tumors, including human breast, brain, colon, and ovarian cancer (Zhou et al., 2009), as well as for categorizing breast cancer molecular subtypes (Hergueta-Redondo et al., 2008; Nakshatri et al., 2009; Stagg and Pommey, 2009). In contrast, the differentiation marker CK18 is highly expressed in the normal mammary glands (more than 90% cells), and its loss has been correlated with high tumor grade (Woelfle et al., 2004). These findings clearly show that cisplatin treatment influenced gene expression in the breast cancer cell lines tested to shift them toward a more differentiated phenotype.

The current anti-cancer drug therapies are undergoing a huge shift from cytotoxic-based to differentiation-inducing therapies, as many types of tumors acquire further resistance, recur, and/or metastasize after treatment due to survival of the resistant CSCs (Prat et al., 2010). The general rule of tumor cell differentiation therapy is to force maturation of less differentiated CSCs or cancer progenitor cells into specific lineages, which in turn reduces proliferation capacity and tumorigenicity (Hadnagy et al., 2008). Based on our findings, we propose that cisplatin not only induces symmetrical differentiation, but also asymmetrical differentiation, shifting BT-549 cells toward a myoepithelial phenotype, and MDA-MB-231 and MDA-MB-468 cells toward a luminal phenotype. The fate of switching from one cell type to another (myoepithelial to epithelial cell type and vice-versa) may depend on the more dominating or prominent progenitor cell type present in each cell line. However, extensive study is required to further confirm the mechanism of cisplatin-mediated induction of differentiation.

**Figure 7:**
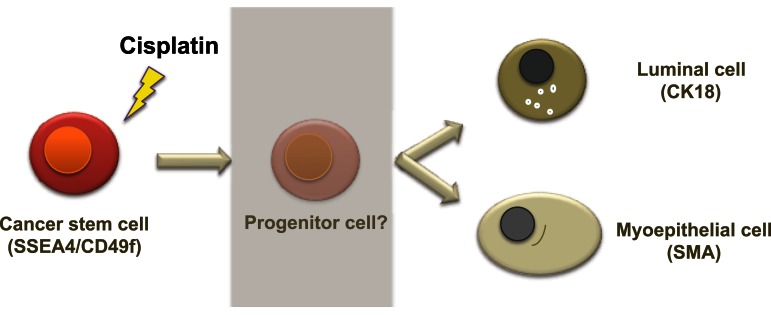
**A proposed model of cellular hierarchy in breast cancer cells and how it is influenced by cisplatin**. The diagram illustrates a possible mechanism of cisplatin effects in breast cancer cells. Cancer stem cells can differentiate, potentially through progenitor steps, into a more mature epithelial phenotype, luminal, or myoepithelial, which has lost or possesses limited proliferative potential. As the more differentiated cells are more susceptible to chemotherapy, this push of differentiation may assist in the management and/or treatment of breast cancer.

A possible mechanism of cisplatin-induced differentiation in breast cancer cells is direct interference of the cis-platinum compound with the DNA sequence at the reactive nuclear sites, causing conformational changes and inhibiting transcription, which eventually leads to cancer cell death. This can be correlated with various levels of binding capacity to target sites in the nuclear DNA of the tumor cell. Even partial binding of cisplatin to the DNA sequence, 1,3-intrastand GpG crosslink, or other unknown crosslink patterns, can trigger changes in gene expression and cell function. A second mechanism of cisplatin action could involve binding of the cis-platinum compound to non-DNA targets. This is supported by a previous study demonstrating that <1% of the cis-platinum compound binds and form adducts with the nuclear DNA of the tumor cell, while 75–85% of it forms covalent bonds with thiol peptides, proteins, RNAs, and other cellular constituents (Cepeda et al., 2007). Other studies have also shown that platinum compounds can bind to cellular proteins such as the zing-fingers, tubulin as well as actin (Cepeda et al., 2007; Wexselblatt et al., 2012). Consistent with this, we show that cisplatin clearly affects the cytoskeleton through morphological changes as well as clear changes in the cytoskeleton proteins β-tubulin, CK18, and SMA (Figures 3 and 4). Binding of cisplatin to these proteins may lead to structural conformation changes that restrict movement of cell proliferation-controlling transcription factors to the nucleus and attachment to their target promoters (Nguyen et al., 2010). Figure 7 illustrates a proposed model based on our findings which depicts the role of cisplatin in differentiating breast cancer cells.

In addition to effects of cisplatin at the protein level, we demonstrated differential gene regulation at the mRNA level. CD49f and β-tubulin mRNA and protein expression were affected in a similar way by cisplatin in BT-549 and MDA-MB-231 cells, suggesting that cisplatin’s point of interference was at the DNA sequence. However, SSEA4 expression was influenced differently at the mRNA and protein levels by cisplatin treatment, with decrease in protein expression, but an increase in mRNA expression. This suggests that this drug may influence certain genes differentially at the post-transcriptional or post-translational levels without affecting the DNA sequence, which could be linked to epigenetic regulation (Hadnagy et al., 2008). This is further supported by a recent study showing that SSEA markers are among the most epigenetically accessible and can in turn mediate the expression of chromatin remodeling factors that are accountable for the principal epigenetic modifications (Sanges and Cosma, 2010). Taken together, these results suggest that in the tumor environment cisplatin may activate binding of other components to DNA, resulting in disruption of DNA transcription.

## Conclusion

Breast tumors are characterized by a cellular hierarchy, similar to the healthy resting and lactating breast (Hassiotou et al., 2012; Hassiotou et al., 2013a,b), containing CSCs and more differentiated cancer cells, each with different susceptibility toward various anti-cancer drugs. Cisplatin seems to shift this hierarchy toward more differentiated cells that are less proliferative. Although the mechanism of action of cisplatin still remains elusive, we demonstrated differential effects at the mRNA and protein levels for some genes, suggesting involvement of both or either of epigenetic mechanisms and recruitment of other cellular components that influence gene transcription and/or translation. Further studies are needed to give insight into how cisplatin acts on tumor cells, and whether and how it may influence their normal cell counterparts.

## Conflict of Interest Statement

The authors declare that the research was conducted in the absence of any commercial or financial relationships that could be construed as a potential conflict of interest.
